# Supplementary material for: High-Throughput Quantitation of Cannabinoids by Liquid Chromatography Triple-Quadrupole Mass Spectrometry
Source: Molecules. 2022 Jan 24;27(3):742. doi: 10.3390/molecules27030742 (PMC8840290; doi:10.3390/molecules27030742)

Supplementary Materials

Figure S1: MRM of CBDVA, collision energy at 10 for product ion 313.1 m/z and collision energy at 26 for product ion 191 m/z.

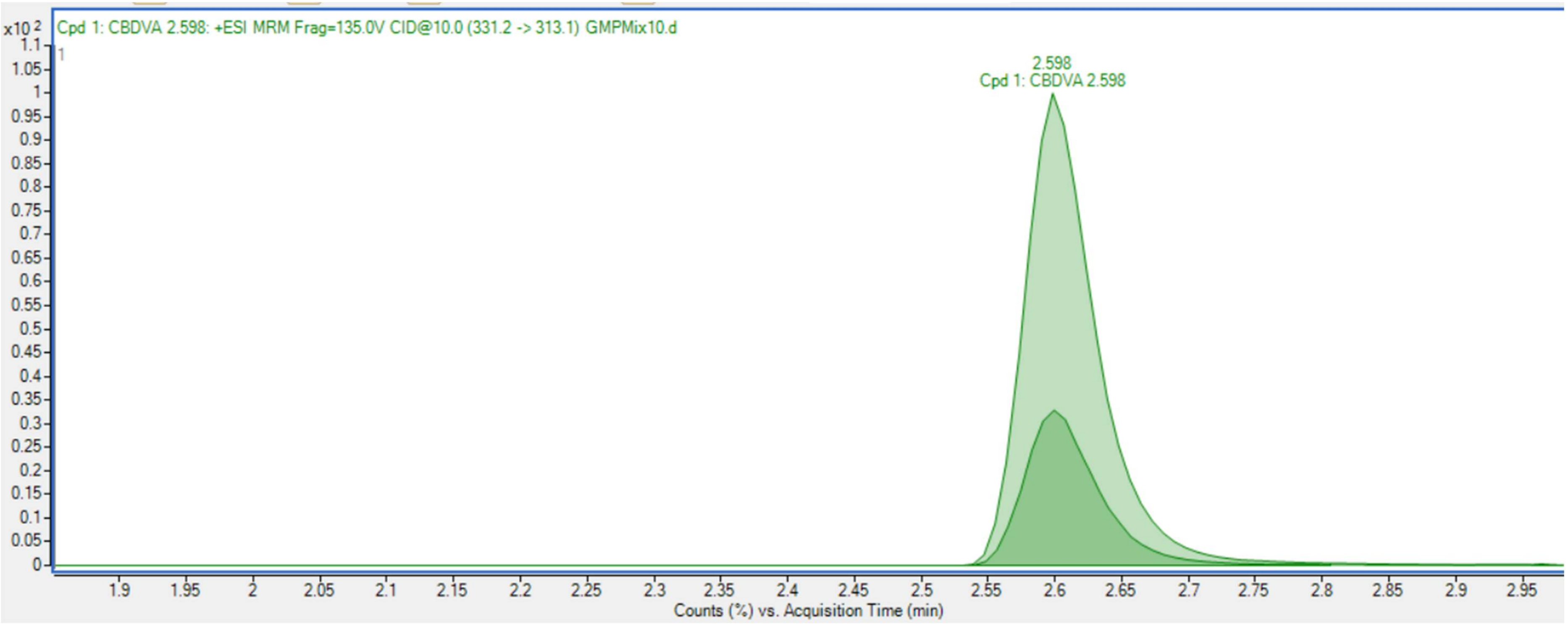

Figure S2: MRM of CBDV, collision energy at 18 for product ion 165 m/z and collision energy at 35 for product ion 123 m/z.

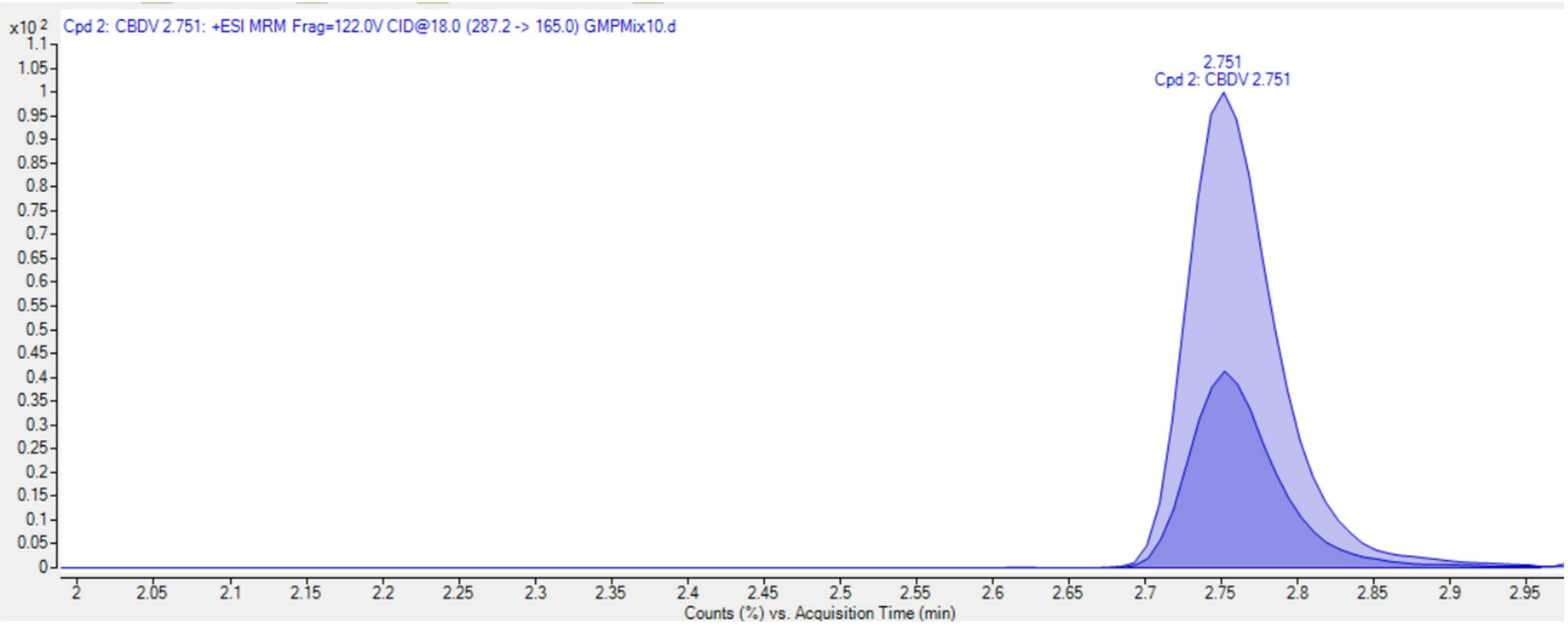

Figure S3: MRM of CBD, collision energy at 10 for product ion 341 m/z and collision energy at 30 for product ion 219 m/z.

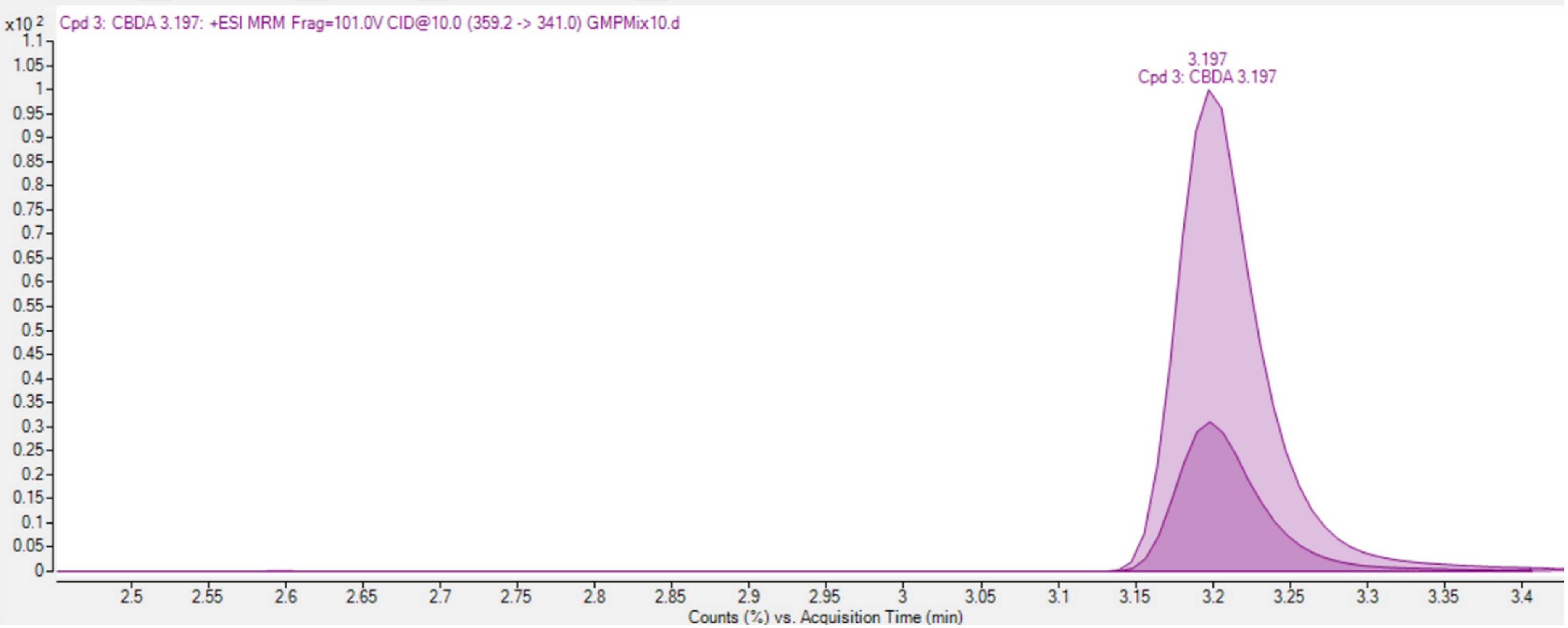

Figure S4: MRM of CBGA, collision energy at 15 for product ion 361.2 m/z and collision energy at 22 for product ion 219 m/z.

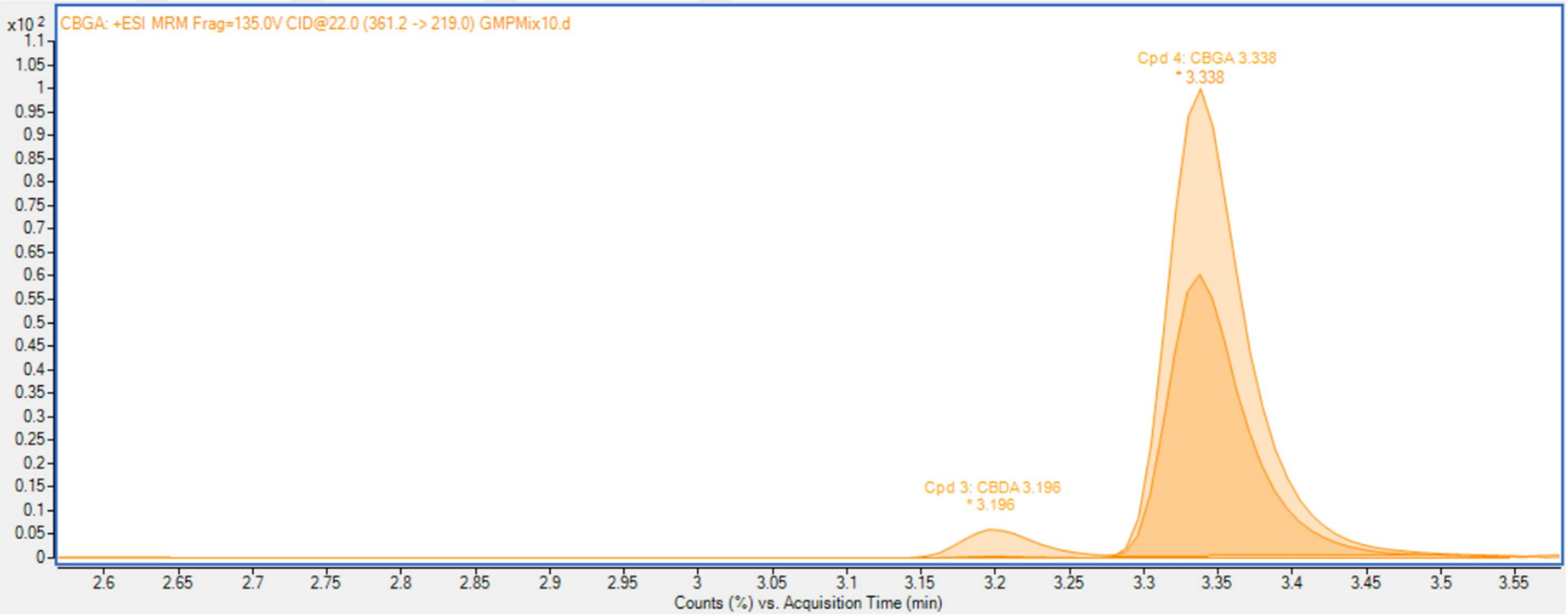

Figure S5: MRM of CBG, collision energy at 10 for product ion 193 m/z and collision energy at 35 for product ion 123 m/z.

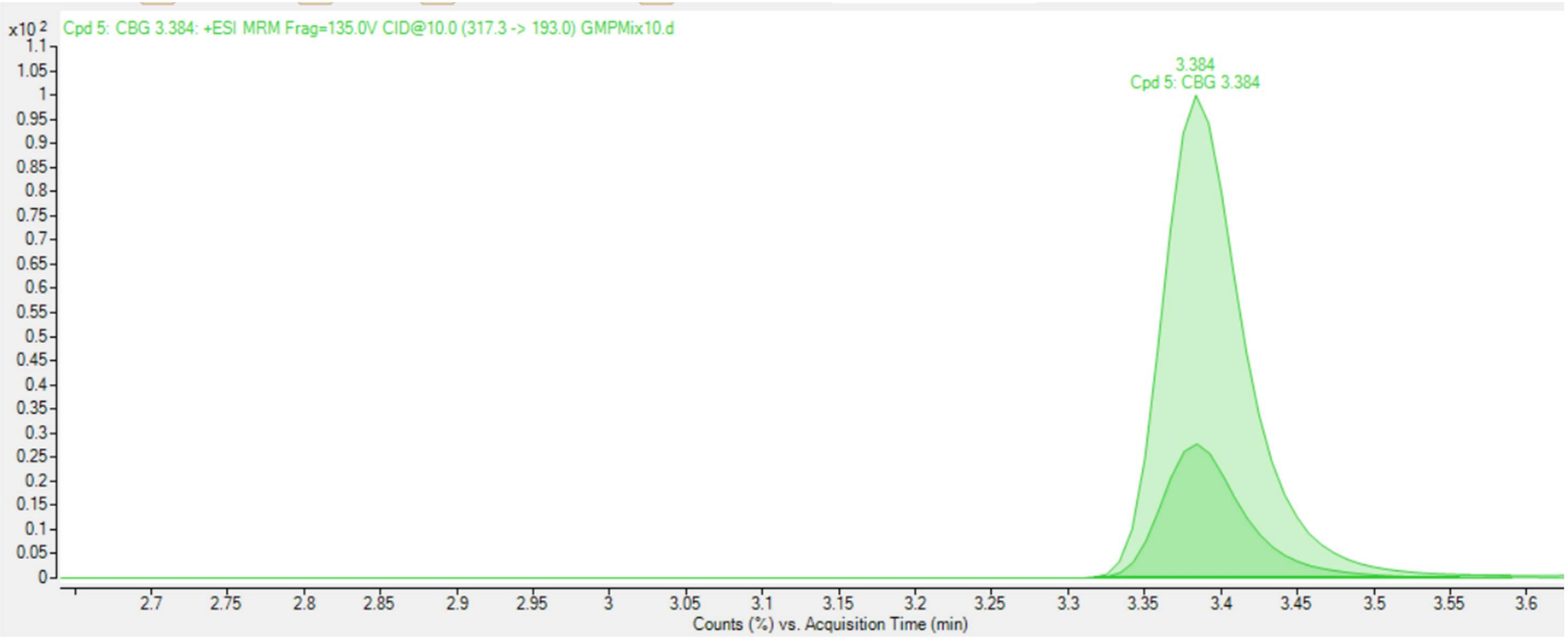

Figure S6: MRM of CBD, collision energy at 18 for product ion 193.1 m/z and collision energy at 30 for product ion 123 m/z.

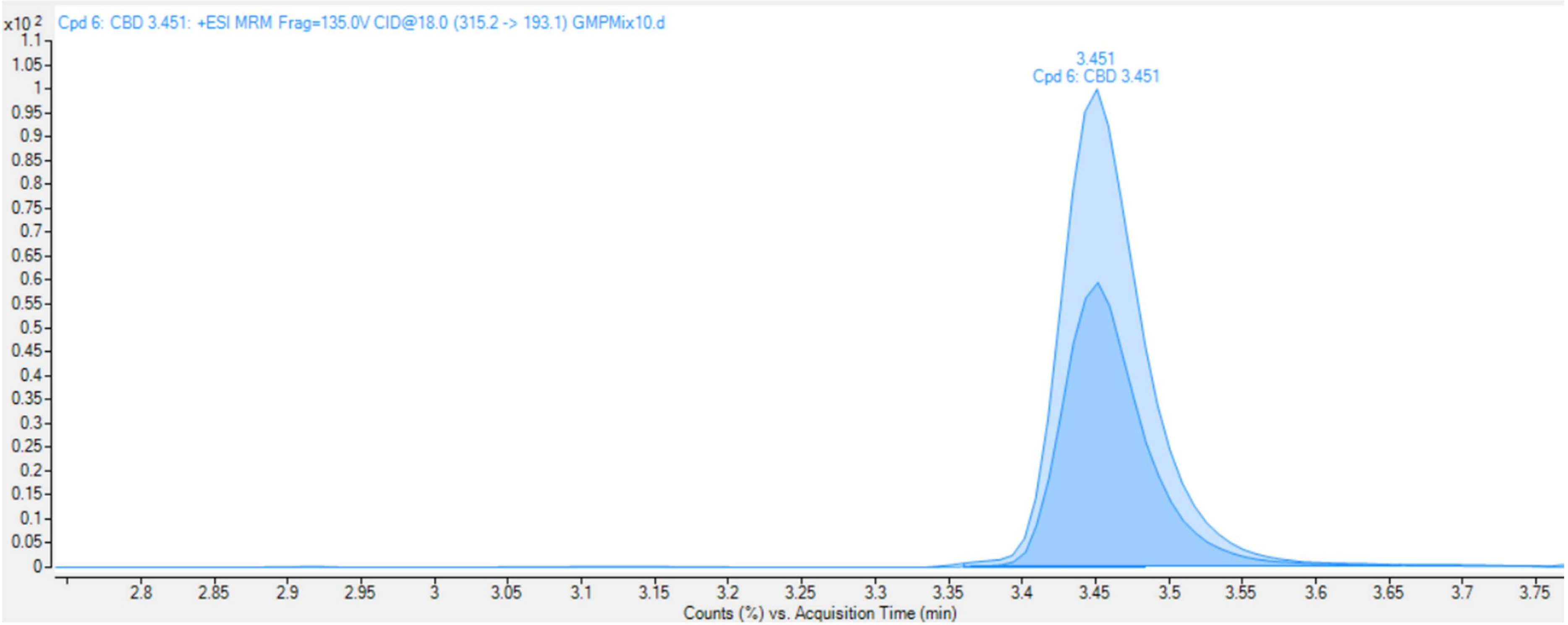

Figure S7: MRM of THCV, collision energy at 18 for product ion 165 m/z and collision energy at 30 for product ion 123 m/z.

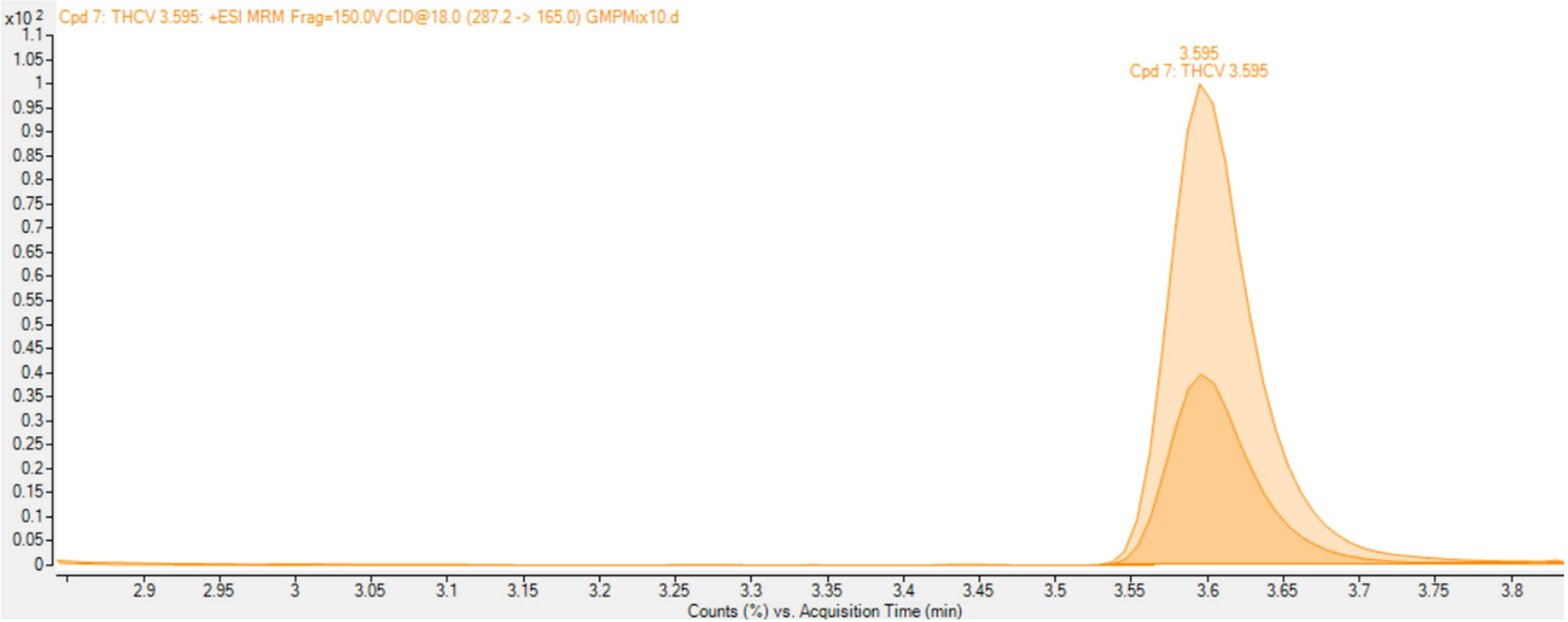

Figure S8: MRM of THCVA, collision energy at 10 for product ion 313.1 m/z and collision energy at 30 for product ion 193 m/z.

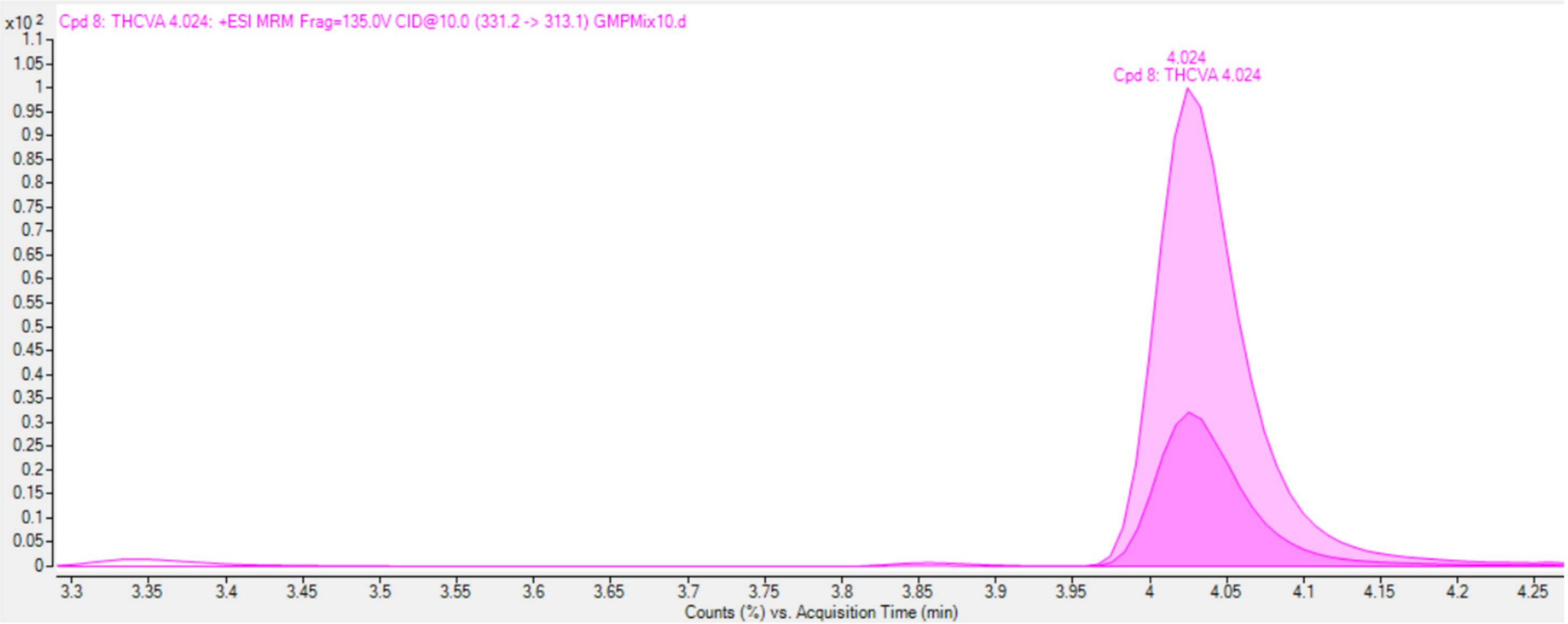

Figure S9: MRM of CBN, collision energy at 18 for product ion 193.1 m/z and collision energy at 30 for product ion 123 m/z.

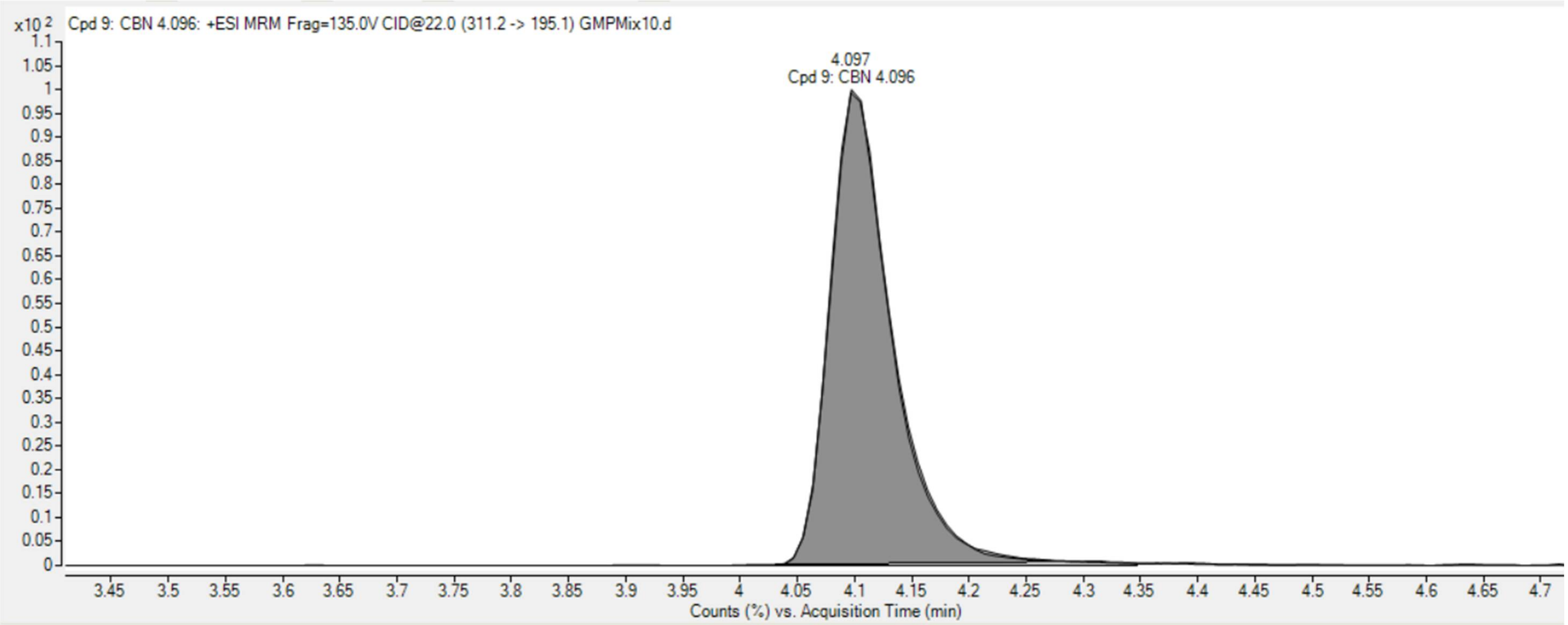

Figure S10: MRM of Δ8-THC, collision energy at 35 for product ion 159.7 m/z and collision energy at 30 for product ion 119 m/z.

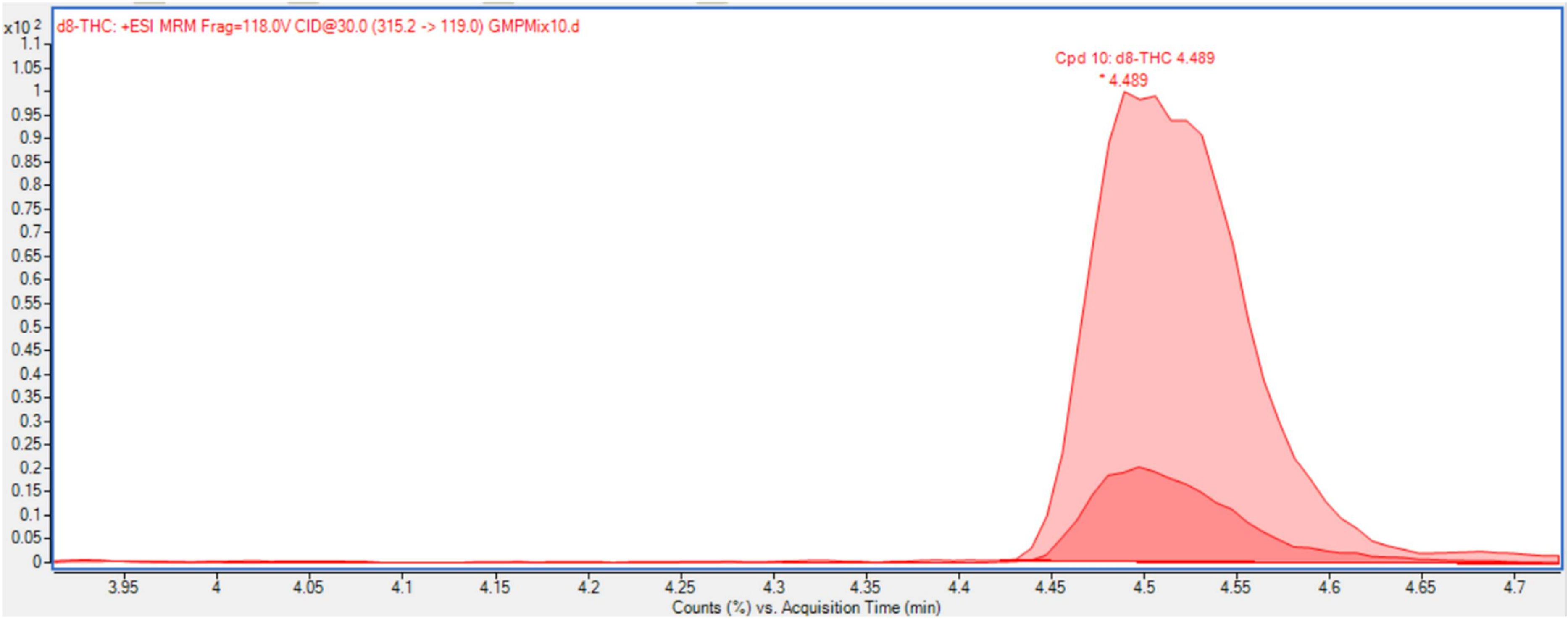

Figure S11: MRM of CBNA, collision energy at 10 for product ion 337.1 m/z and collision energy at 30 for product ion 235.1 m/z.

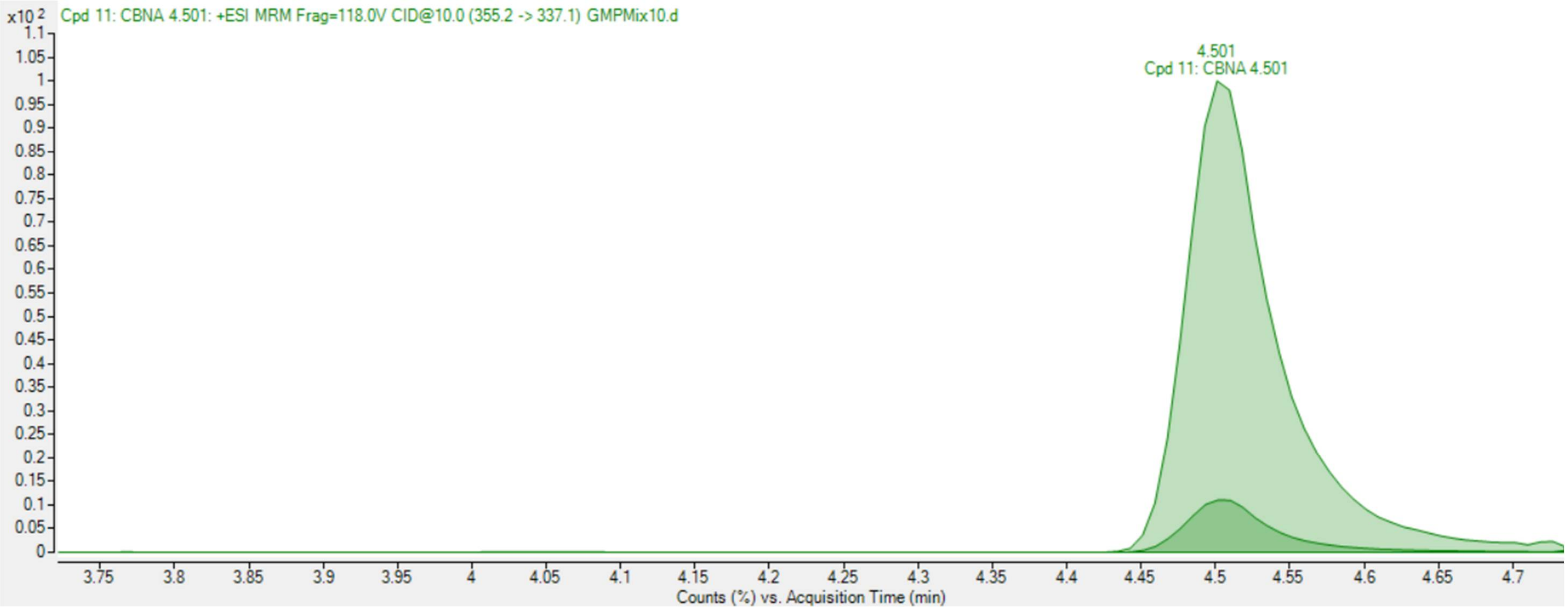

Figure S12: MRM of CBC, collision energy at 18 for product ion 217 m/z and collision energy at 20 for product ion 259 m/z.

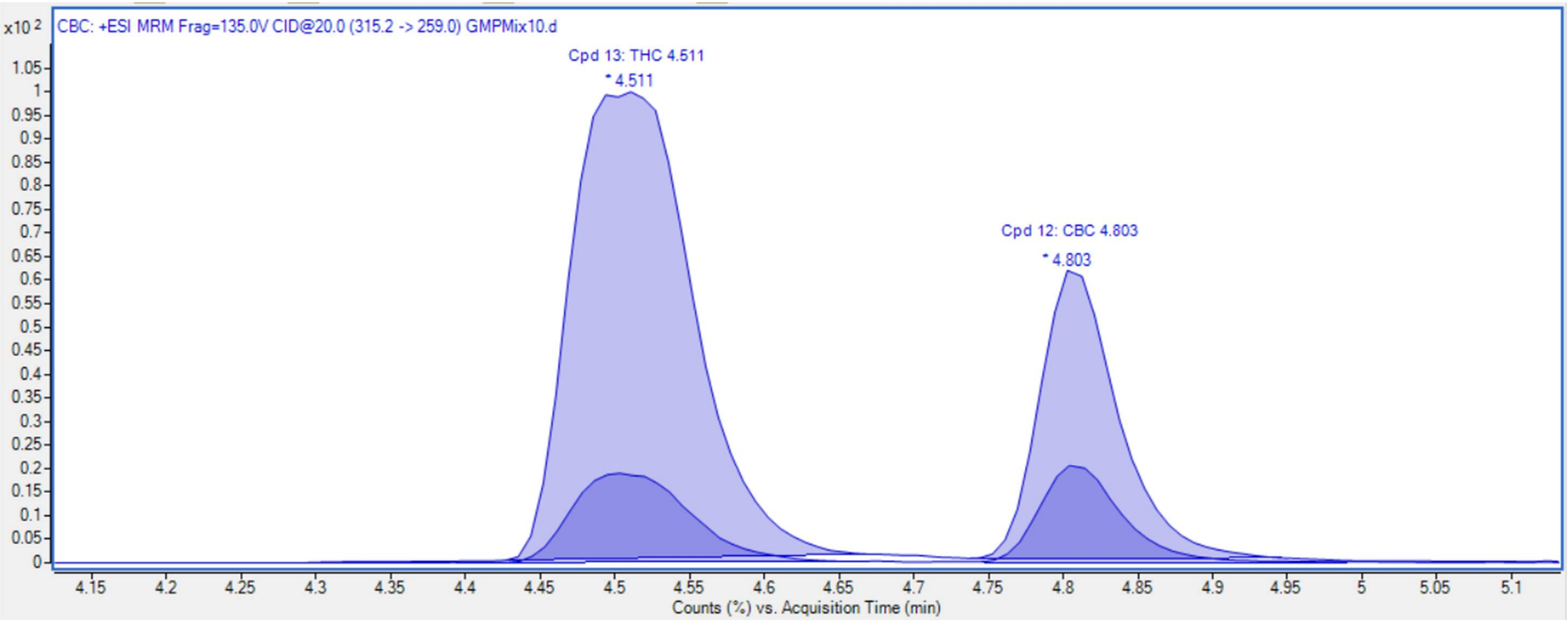

Figure S13: MRM of THC, collision energy at 22 for product ion 217 m/z and collision energy at 22 for product ion 165.1 m/z.

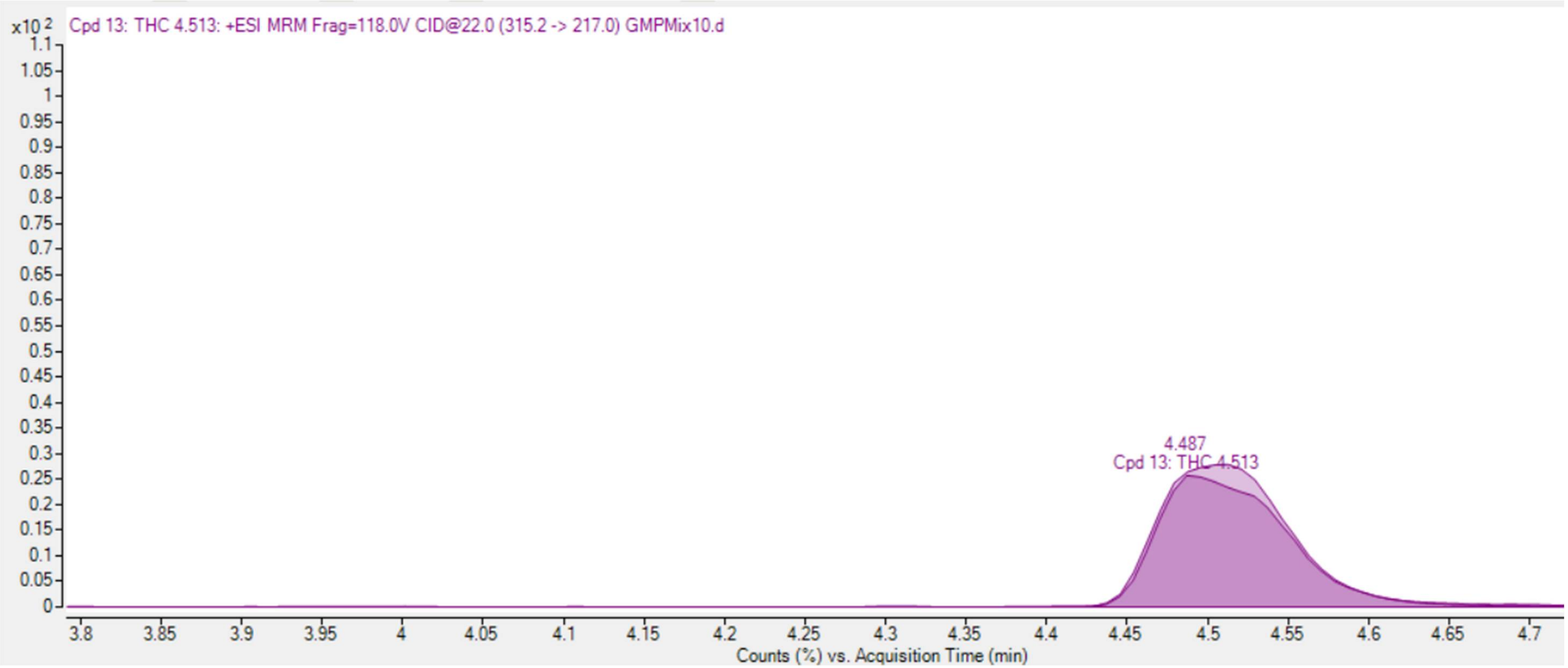

Figure S14: MRM of CBL, collision energy at 20 for product ion 235.1 m/z and collision energy at 26 for product ion 165 m/z.

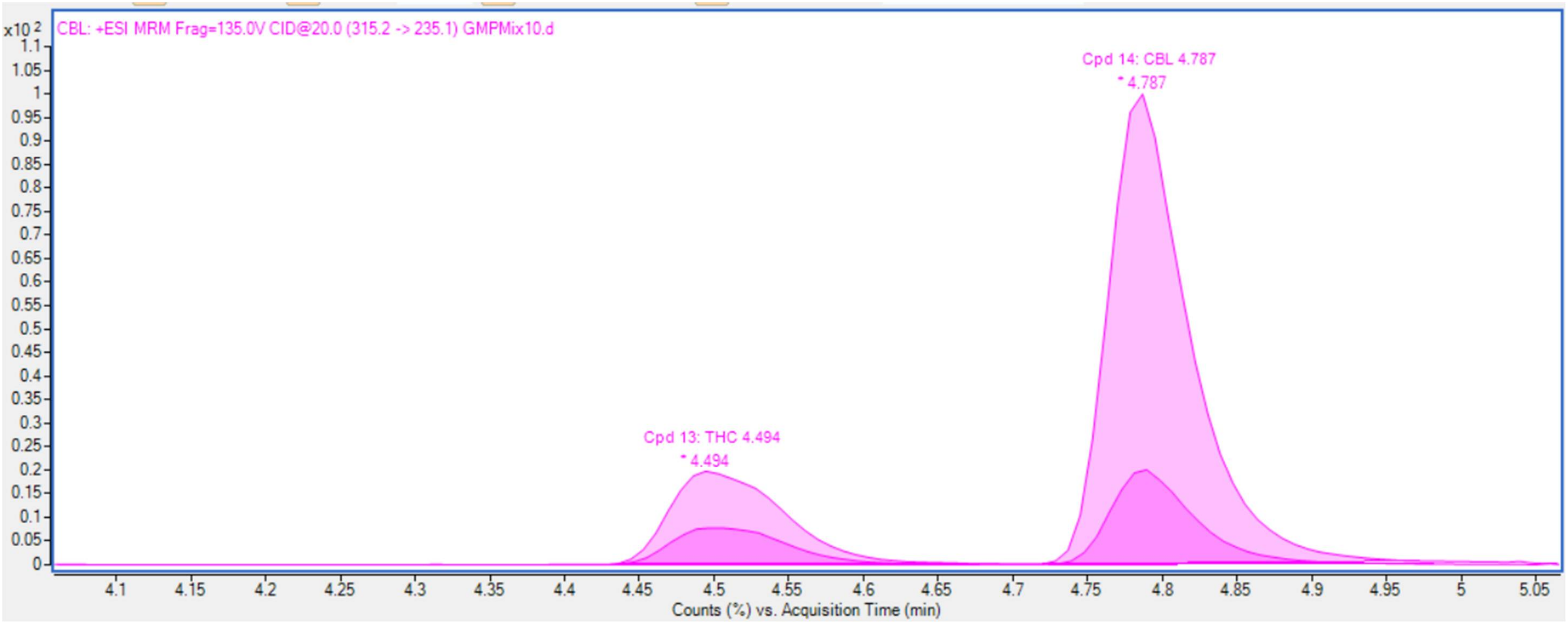

Figure S15: MRM of THCA-A, collision energy at 6 for product ion 341.1 m/z and collision energy at 26 for product ion 219 m/z.

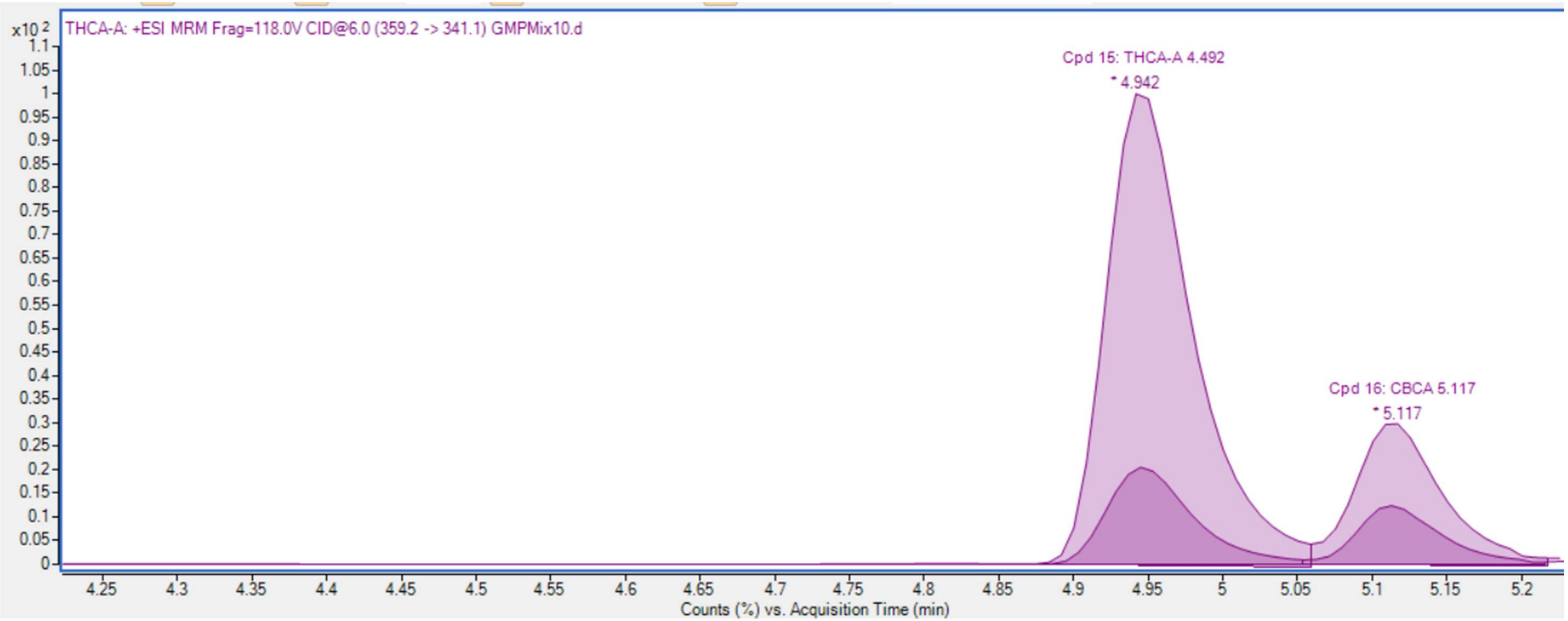

Figure S16: MRM of CBCA, collision energy at 18 for product ion 233 m/z and collision energy at 18 for product ion 219 m/z.

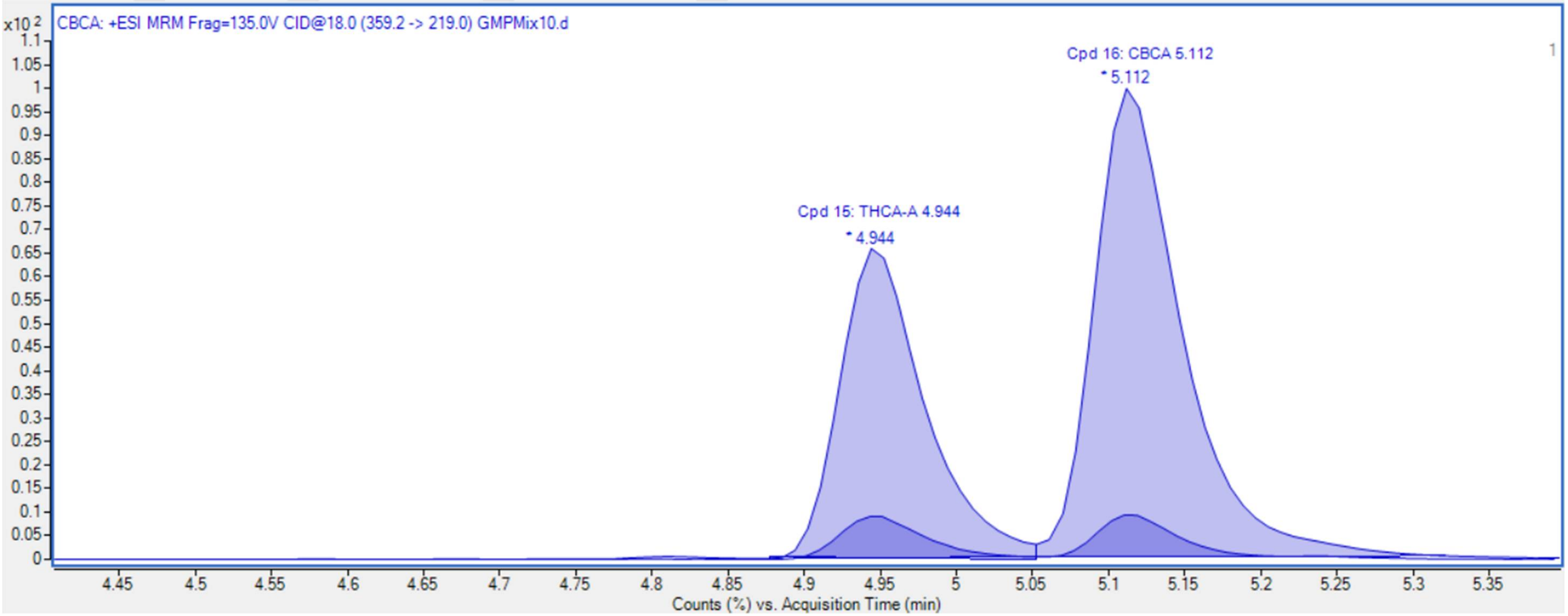

Supplement: Supplementary file 1 [file molecules-27-00742-s001.zip › molecules-1488739-supplementary.pdf]
